# The use of care home environments to meet culture-specific needs of culturally and linguistically diverse residents with dementia: an integrative review using the ICF framework

**DOI:** 10.1186/s12939-025-02748-0

**Published:** 2026-01-16

**Authors:** Nina Ramezani, Sarah Granberg, Annica Kihlgren, Katarina Baudin, Helen Lindner

**Affiliations:** 1 School of Health Sciences, Örebro University, Örebro, Sweden; 2 The National Agency for Special Needs Education and Schools, Örebro, Sweden; 3 Division of Occupational Therapy, Department of Neurobiology, Care Sciences and Society, Karolinska Institutet, Huddinge, Sweden

**Keywords:** Alzheimer disease, Dementia, Cultural diversity, Ethnicity, Ethnic and racial minorities, Environment, Integrative review

## Abstract

**Background:**

Increasing global migration creates new challenges for multicultural societies in providing equitable care. Culturally and linguistically diverse (CALD) people who move into care homes find themselves in an environment where health professionals do not speak their language and the access to cultural activities is limited. This may increase loneliness and social isolation. When designing care home environments for CALD residents with dementia, culture is a key consideration. The aim of this integrative review is to highlight *what* elements of the care home environment are reported to meet culture-specific needs of CALD residents with dementia, and *how*.

**Methods:**

A search strategy which included terms for care homes, forms of dementia and CALD people was developed, and a systematic search was carried out in six databases. Eligible articles were original peer-reviewed studies published between 2013 and 2024 and contained examples of how care home environments have been used to meet culture-specific needs of CALD residents. All screenings and extractions were carried out by two independent researchers.

**Results:**

The search resulted in 4311 records. After the screening process, 27 articles met the eligibility criteria. The review findings are categorized according to components of the WHO’s International classification of functioning, disability and health (ICF). Results linked to the ICF component *Activities and participation* stress the importance of communication in the resident’s preferred language, social and supportive relationships and culturally relevant activities, while the component *Environmental factors* highlights the significance of ethnic food and support from culturally competent care professionals and family members.

**Conclusions:**

This integrative review underlines the complexity of using environments to meet culture-specific needs of CALD residents with dementia. The findings highlight the importance of bilingual staff, culturally relevant activities and inclusive environments in enhancing communication, building interpersonal relationships and reducing frustration among CALD residents. Collaborations between culturally competent staff, family members and members of cultural communities also facilitate meeting social and cultural needs of these residents. This review offers suggestions on how environments in care homes can be adapted for CALD residents and encourages further research to find practical solutions for equitable care.

**Registration:**

A study protocol is registered on Prospero (CRD42023492906).

**Supplementary Information:**

The online version contains supplementary material available at 10.1186/s12939-025-02748-0.

## Introduction

Roughly 57 million people worldwide live with dementia, making it one of the greatest causes of dependency among elders globally [1]. Dementia is a hypernymy for several disorders that are all neurodegenerative and have behavioural, psychological and cognitive symptoms such as irritability, anxiety, disorientation and memory loss [1, 2]. As the disorder progresses the decline in cognitive functions may become severe enough to interfere significantly with activities of daily living (ADLs) [1]. Persons with dementia may experience difficulties in performing both instrumental ADLs (e.g. cooking, shopping, managing medications) and basic ADLs (e.g. bathing, dressing, toileting), leading to increased dependence on others and reduced quality of life [3, 4]. As a result, it may eventually become necessary for the person with dementia to move into a care home.

Care homes are typically set up to remind their residents of home, creating a familiar setting for the majority population of the country they are in. With increasing global migration, however, more people are living in countries where they were not born and whose language they are not proficient in. These individuals are often referred to as culturally and linguistically diverse (CALD) people or populations [5, 6]. In Europe, nearly 6.5% of people with dementia are CALD older adults [7]. These percentages are expected to increase in Europe and worldwide due to ongoing migration [8, 9]. As dementia progresses, CALD people may lose their second or other languages, returning predominantly to their mother tongue [10]. This can be problematic in care homes as health professionals may not speak the preferred language of the person living with dementia, and the access to relevant cultural and religious activities may be limited. Scarcity of interpersonal relationships or activities could result in increased loneliness and social isolation, which in turn may accelerate the disease’s progression [11]. Multicultural societies therefore face a new challenge of securing equitable care for culturally and linguistically diverse (CALD) persons with dementia within care homes.

The environment of care homes is widely recognized for its significant impact on the well-being of residents with dementia [12–14]. When designing care home environments for CALD residents with dementia, culture is a key consideration, as it can serve either as a therapeutic resource or a therapeutic barrier in their care [15]. Culture refers to the traditions, beliefs, values, and lifestyles shared by a group of people. It includes several elements such as religion, gender perspective and views towards illness [16]. Recent reviews of studies taking place in care homes have highlighted culture-specific needs of CALD residents, such as a shared language for communication, access and presentation of traditional food, activities and spaces that promote cultural and religious practices [17–20]. These needs are often unmet in care homes, which may intensify dementia-related behavioural symptoms. A care home environment with elements that are culturally familiar to CALD older adults fosters a sense of belonging and promotes well-being [18]. A comprehensive review of how to meet these culture-specific needs is therefore needed.

In 2001 all 191 member states of the World Health Organization (WHO) endorsed a newly developed *International classification of functioning*,* disability and health* (ICF) [21]. Since the diagnosis alone is not enough to predict care needs or functional outcomes, the ICF works as a standardised language to describe health and health-related components of well-being through components of functioning and disability [22]. Recognising that function and disability is also directly affected by contextual factors, environmental factors were also included in the classification system. Environmental factors can be both physical, social, attitudinal or organisational elements surrounding a person [22]. The ICF has since been used in both clinical settings and in research to describe dementia-related functioning and disability to create a better understanding of persons living with dementia [23]. The ICF has also been used to develop methods of analysis of health-related data [24]. Given that the ICF highlights the impact of environmental factors on health and well-being and provides a systematic system for coding and analysing such factors, it was deemed suitable to detangle the complex phenomenon at the centre of this integrative review.

The WHO also encourages person-centred care (PCC) for all care related situations involving people with dementia [25]. PCC promotes a shift from treating the patient as a passive recipient of care, to them being an active partner in the decision-making process involving their own care [26]. Yet, definitions of PCC often disregard the importance of designed, physical environments [27]. Currently, no review of the use of care home environments to meet culture-specific needs of CALD people with dementia has been identified. The aim of this review is therefore to:

- Highlight what elements of the care home environment are reported to meet culture-specific needs of CALD residents with dementia.
- Describe *how* these elements of the care home environment have been used in relation to CALD residents according to previous research.

## Methods

### Design

The integrative review methodology [28] was selected for this study to allow for the inclusion of original studies of diverse research methodologies such as qualitative, quantitative and mixed methods. This review methodology also enables a wide scrutiny of a defined phenomenon, which is suitable for the aim of this review. The review process consisted of the following stages: (1) problem formulation, (2) literature search, (3) data evaluation, (4) data analysis, (5) presentation of data. A study protocol for this review was registered on Prospero (CRD42023492906).

### Search strategy

A comprehensive systematic search [28] was carried out in six databases (Applied Social Sciences Index and Abstracts [ASSIA], Cumulated Index to Nursing and Allied Health Literature [CINAHL], MedLine, PsycINFO, Scopus and Web of Science), each selected for its relevance to our research topic, on 20 December 2023. The search terms and search strategy were developed in collaboration with an experienced research librarian to enhance the search quality. Search strategies were adapted to each database’s search requirements. Three search blocks were used, where the first block consisted of search terms for the study setting (care homes), the second search terms for the health condition (dementia) and the third search terms for the population (culturally and linguistically diverse people). These search blocks were combined at the last step (Appendix 1). Each block contained both subject headings and free-text terms, combined using Boolean operators. The relevance of search terms was tested through scoping searches where the impact of the term’s addition and the relevance of its outcome was examined. Searches were initially limited to the years 2013–2023, but a complementary search was carried out on 18 December 2024 to include more recent research. A manual search in the reference lists of eligible studies was also carried out.

### Eligibility criteria

In order to further narrow the search to fit the aims of this integrative review, the following inclusion criteria were established:

- Studies where the use of physical, social, attitudinal or organisational environments in care homes can be distinguished.
- Published in peer-reviewed scientific journals.
- Original studies.
- Published 2013–2024.

The exclusion criteria were the following:

- Not specified to culturally and linguistically diverse residents.
- Not specified to people with dementia.
- Studies conducted only in the community, hospice or acute care settings.
- Studies focused on the development and validation of new instruments or assessments.
- Reviews, study protocols, commentaries, editorials, feasibility studies, pilot studies.

Pilot and feasibility studies were excluded since these are often used to test an idea or the feasibility of an intervention, while this integrative review aimed to seek studies that provided more developed evidence of how environments were used to meet the culture-specific needs of CALD residents in care homes.

### Study selection

Study selection was carried out in the systematic software Covidence [29], which helped identifying and removing duplicates before the screening commenced. The inclusion and exclusion criteria were applied during the screening of titles and abstracts which was carried out independently by two researchers (H.L. and N.R). Any conflicts of inclusion or exclusion of an article were resolved by researcher K.B. The full-text screening was carried out by H.L. and N.R. independently. Any conflicts were resolved by researcher S.G. All remaining full-text articles were then once again discussed in detail by H.L. and N.R. to reach consensus. Finally, a manual search was carried out based on the reference lists of the included articles.

### Quality assessment

The methodological quality of the studies included in this review was assessed using the Mixed Method Appraisal Tool (MMAT) [30]. MMAT is divided into two sections specifically designed for evaluating studies of different study design. The first section consists of two screening questions that verify whether a study employs empirical data. In the second section, each study is rated by selecting the appropriate study design category. Two reviewers, A.K. and H.L., independently assessed the methodological quality of each study. Any disagreements in their evaluations were resolved through discussions until consensus was achieved.

### Data extraction strategy

A data extraction form was first developed based on the findings from recent reviews involving CALD residents with dementia living in care homes [17–20]. The form was then assessed for face validity by K.B. and S.G. The form was then piloted by N.R. and some changes were made accordingly. The final data extraction form included the following headings: author(s), year of publication, study design, method of data collection, study aim(s), care effort/intervention, participants’ characteristics (for example first language / ethnicity), description of care home setting and its health professionals, and main results related to the use of physical, social and organisational care environments to meet culture-specific needs of residents.

### Data analysis

The strategy for data analysis is one of the least developed aspects of the integrative review process [28]. In the current study a simplified content analysis [24] from the perspective of ICF [22] was conducted. ICF provides an opportunity to organize data in a structured manner and provides possibilities to, specifically, code environmental aspects. Although ICF consists of four components, only coding in relation to *Activities and participation (d)* and *Environmental factors (e)* were included. The component *Activities and participation*, in this paper, contains elements that have a clear connection to culture-specific needs in the care home environment, such as relationships, social and community life and communication. Meanwhile, *Environmental factors* contains information about culture-specific physical, social and organisational environments. The extracted data was systematically coded in full by N.R. using a linking extraction table (Table 1). In this table each identified meaning unit from the included studies was broken down to identify the meaningful concepts, interpret the underlying meaning and extracting linking units. To connect, *link*, these units to specific ICF categories, the ICF linking rules by Cieza et al. [31] were used. These linking rules have been developed to ensure a uniform method for linking information to ICF categories and strengthening the reliability of the linking process. Once N.R. had finished coding all data, the coding of 50% of the studies, chosen through random selection, was examined by S.G. who made suggestions for revisions. N.R. then re-examined all the coding and made appropriate revisions.

**Table 1:** Example of content analysis of the extracted data using a linking extraction table

1. Meaning unit	2. Meaningful concept	3. Interpretation of underlying meaning	4. Linking unit	5. ICF category	6. Linking rule
“Sometimes she doesn’t understand what we are telling, but when [her Italian speaking friend] comes, when she speaks in Italian, she sometimes calms down. You know, she speaks to her.”	When [her Italian speaking friend] comes; she speaks and sometimes calms down.	Having a friend who speaks the CALD resident’s native language sometimes calms her down.	Italian speaking friend Communication Calming effect	e320 Friends d350 Conversation d7202 Regulating behaviours within interactions	Cieza et al., rule 2 Cieza et al., rule 3 Cieza et al., rule 6

The seventh step (not displayed above) of the linking extraction table encourages the researcher to verify the selected ICF categories in relation to the original meaning unit

## Results

**Fig. 1:**
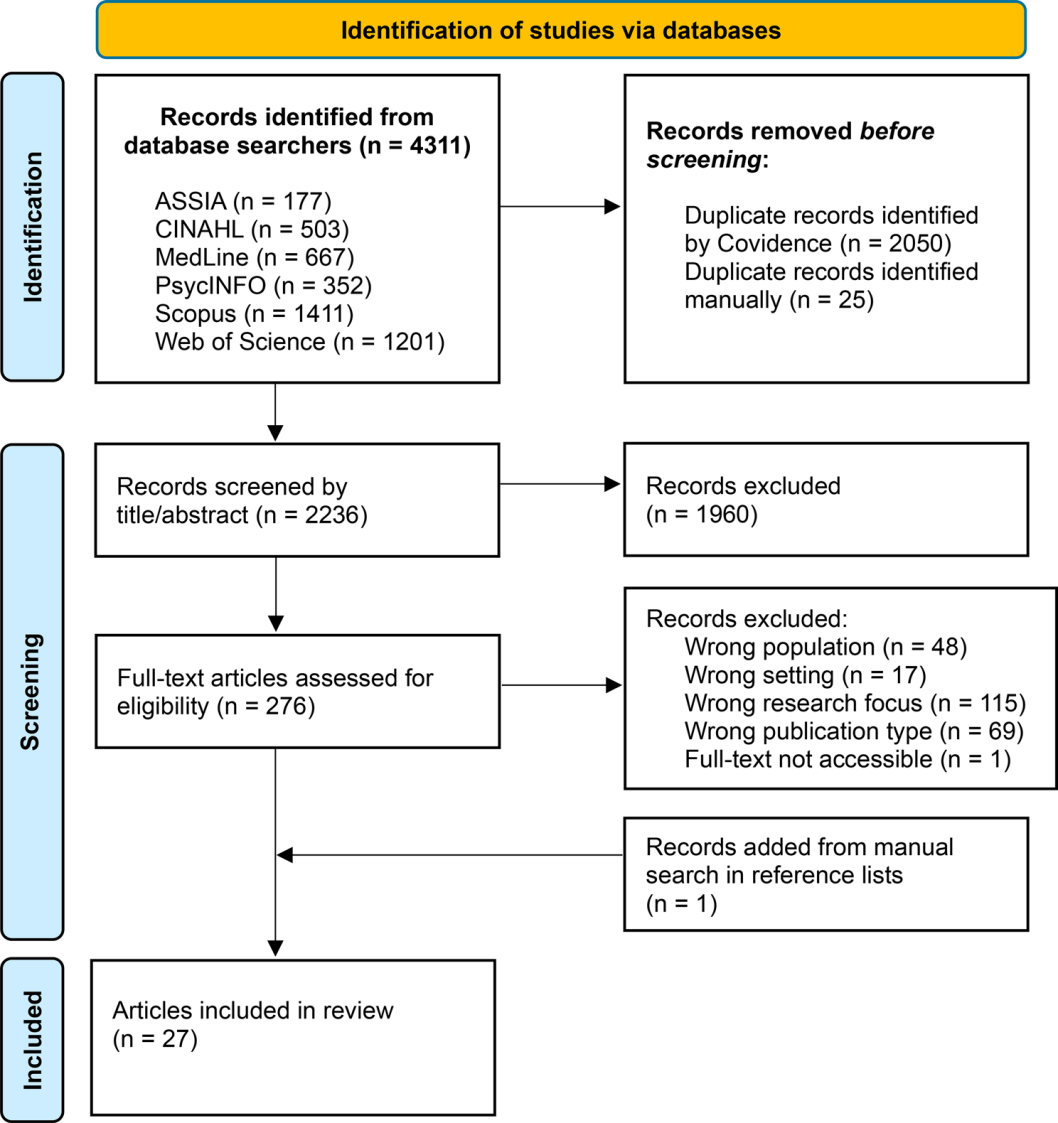
Preferred reporting items for systematic reviews and meta-analyses (PRISMA) flow diagram of the selection process

The database searches generated 2236 unique studies. 1960 articles were excluded through title and abstract screening and a further 250 articles through full text screening, leaving 26 studies. A screening of these articles’ reference lists led to the inclusion of one additional article. The search outcome is summarised in a PRISMA flow diagram [32] in Fig. 1. After quality appraisal it was decided that no studies should be excluded due to low methodological quality. See Appendix 2 for the detailed ratings using MMAT. The final data set contained 27 studies (Appendix 3) using qualitative (*n* = 21), quantitative (*n* = 3) and mixed method (*n* = 3) study designs. The studies were carried out in 10 different countries: Sweden (*n* = 8), Australia (*n* = 7), Canada (*n* = 3), South Africa (*n* = 3), The United States of America (*n* = 3), Norway (*n* = 2), The United Kingdom (*n* = 2), The Netherlands (*n* = 1), New Zealand (*n* = 1) and Singapore (*n* = 1), where a few studies were performed in more than one country. Seven studies contained data collected in ethno-specific or culturally profiled care homes, in other words care homes that were specifically designed for a specific group of residents that belong to an ethnic minority in their country of residence.

The extracted data, which was coded in accordance with ICF within the components *Activities and participation (d)* and *Environmental factors (e)*, is summarised below. It is important to note that the results contain the points of view of both residents, relatives, health professionals, people in leadership positions and researchers. While some results describe how environments have been used in the participating care homes, others describe what these groups of people describe as lacking in their environment. These perspectives together highlight what aspects of the care home environment are reported to meet the culture-specific needs of CALD residents with dementia and describe how these have been used.

### Activities and participation (d)

The ICF component *Activities and participation* describes the ability or opportunity a person has in carrying out a task or an action, and the person’s involvement in life situations [22]. The coded findings are all from the perspective of the person whose life situation is being described – in this case the CALD resident with dementia. While the extracted data was originally coded as detailed as possible, often using third level codes (d9202 – Arts and culture, d9205 - Socializing), the narrative description of the results is presented at the first level/chapter level (d9 – Community, social and civil life) in order to display clearer themes. The data was linked to a total of 29 different ICF categories within this component. The most frequently used categories are presented using second level codes (d920 – Recreation and leisure) in Table 2, to illustrate what categories were found to be most significant based on the coded data. The table’s occurrences column indicates how many out of the 27 included studies were linked to that specific ICF category.

#### Communication (d3)

Findings linked to this chapter contained information about communication possibilities from the CALD resident’s point of view. There were examples of where CALD residents were given the opportunity to speak their own language and obtain linguistic stimulation [33–40]. This was often through conversations with bilingual health professionals or with related or unrelated individuals visiting from outside the care home setting [33, 41]. The presence of language barriers [37–39, 42–50] was, however, also a common theme due to the absence of health professionals that shared the resident’s language. For example, one study described how residents who asked for more food were led away from the table because health professionals interpreted it as them being full [51]. Language barriers could also result in CALD residents lacking linguistic stimulation [38] in the care home. Residents were described to communicate with health professionals using non-verbal communicative strategies such as body language [44, 52] or with the assistance of cue cards [50].

#### Interpersonal interactions and relationships (d7)

The communicative theme extends to the importance of creating opportunities for CALD residents to form interpersonal relationships. While some studies found that relationships with health professionals and other residents were formed across linguistic and cultural barriers [34, 41, 43, 44, 46, 50, 52–54], others found that the presence of someone who spoke the residents’ preferred language was beneficial. Interactions in the resident’s native language were shown to have a calming effect on the resident, decrease levels of frustration, and increase their level of activity [33, 38, 42]. Culturally profiled care homes and unprofiled care homes both stressed the benefits of having bilingual health professionals who shared language abilities with the CALD resident among their staff [44, 49], as these had an advantage in interacting and building informal relationships with CALD residents. The presence of other residents with shared cultural and linguistic backgrounds was also beneficial in facilitating interpersonal interactions and forming relationships [38, 39, 54, 55]. At care homes where none of the health professionals or residents shared a language with a specific CALD resident, their family relationships became crucial [34, 37, 38, 43, 50]. Relationships could also be formed with bilingual volunteers [34, 35, 44] or members of cultural organisations [43].

#### Community, social and civic life (d9)

Culturally significant activities enable CALD residents to feel closer to their own community – giving them a sense of home. This could be activities such as watching culture-specific TV programmes [36–38, 48, 50], films [43, 54] or sports [43]. It could also be listening to culture-specific radio programmes [36, 38], reading culture-specific newspapers [38] or playing culturally significant boardgames [43]. Some studies give specific examples of cultural activities for residents of a certain ethnicity, such as smoking the waterpipe [36] for a Persian resident, taking a sauna [38] for a Finnish resident or listening to yoiking (a traditional form of singing in Sami culture) for a Sami resident [51]. However, residents of minority backgrounds in unprofiled care homes sometimes felt like culturally relevant activities had to be restricted to their own private rooms to not disturb other residents, which increased the risk of social isolation [38, 48].

Being part of a larger culturally sensitive community was something which seemed to benefit CALD residents. Sometimes a sense of community could be reached through securing proximity to people of shared cultural heritage from within or outside of the care home [36, 39, 43, 44]. To organise events where CALD residents could socialise with other residents of the same linguistic and cultural background, immediate family, volunteers or members of cultural organisations has therefore been shown to be beneficial. Such events could be organised religious activities [40, 43, 50] or culture-specific celebrations and holidays [36, 38, 39, 43, 44, 50, 55]. These culture-specific celebrations could sometimes be held at the care home. Their elements of culture-specific music, dance and singing sometimes reached across linguistic and cultural barriers to encourage health professionals and residents from mixed backgrounds to participate in the celebrations [34, 43, 50].

**Table 2:** Overview of main findings related to activities and participation

ICF code	Category	Variation within code	Occurrence *n* = 27
d350	Conversation	This code gathers meaningful concepts related to communication possibilities for the CALD resident. This may include information about who the residents converse with, but also information about communicative strategies when the resident does not share a verbal language with the health professionals.	20 (74,1%)
d750	Informal social relationships	This code gathers meaningful concepts related to opportunities for CALD resident to form informal social relationships with people in their surroundings and who these people may be.	18 (66,7%)
d920	Recreation and leisure	This code gathers meaningful concepts related to activities CALD residents enjoy engaging in. This may include both social and solitary activities with cultural relevance.	20 (74,1%)
d930	Religion and spirituality	This code gathers meaningful concepts related to all types of religious activities. This may include both organised religion and private religious practices.	10 (37,0%)

### Environmental factors (e)

The ICF component *Environmental factors* include all physical, social and attitudinal environments of the care home in which CALD residents with dementia live and conduct their lives [22]. As a result, the findings below do not describe, for example, the communication strategies or attitudes of the CALD residents, but rather those of people surrounding the resident. Once again, the narrative description of the results is presented at first level/chapter level, while the most frequently used ICF categories within the chapter are presented at the second level in Table 3 in order to offer more detail. In total, the data was linked to 23 different ICF categories within this component.

#### Products and technology (e1)

This ICF chapter contains a broad array of natural or human-made physical products or systems of products that range all the way from consumables to furniture and technical products with different areas of application. The most commonly occurring element was access to ethnic food and drinks. These findings describe both the residents’ preference for and the positive impact of such food and drinks [33, 36–38, 47, 51, 55, 56], but also the care homes’ difficulty in providing them due to meals usually being adapted to the majority population’s preferences and staff lacking sufficient knowledge about other food traditions [40, 48, 50, 56]. The results therefore describe how ethnic food is sometimes provided through the personal initiatives of family members [38, 43] or visiting volunteers that share the resident’s cultural background [35]. Other important elements with cultural significance were access to a furnished prayer room or chapel for religious practice [36, 40, 50] and furniture [36] or decorations [35, 36, 43, 44, 51] such as Persian carpets or origami, that could further make CALD residents feel a sense of belonging. These elements seemed more frequently available in culturally profiled care homes, compared with mainstream care homes.

This chapter also included products or technology used to assist in communication across linguistic barriers. Health professionals who did not share a language with a CALD resident sometimes relied on the use of language or cue cards, dictionaries or devices with access to Google translate to communicate with them in their native language [38, 43, 44, 50]. Furthermore, a few studies mentioned technology specifically developed to encourage engagement and reminiscence among CALD residents. One such example was dementia-friendly culturally relevant television videos [54]. Such videos came in the resident’s preferred language and were specifically developed for people living with dementia. The content could be ethnic music, displays of cultural events or familiar places that stimulate the CALD residents to connect with their past and share it with others. Another example of technology that encourages engagement in physical and social activities across linguistic barriers was “Touchscreen Technology” and a system called Sitdance [46], a seated dance tutorial for older people. This system successfully engaged residents in dance, creating a social connection between residents and health professionals of all backgrounds.

#### Support and relationships (e3)

Health professionals are presented as the most available social support to CALD residents with dementia living in care homes. Some show interest in learning about the resident’s past experiences [34] and encourage them to participate in activities [46]. There are many instances where health professionals that do not share a language with the resident try to form relationships through alternative communication strategies such as using non-verbal communication, learning key words or phrases in the resident’s language, taking advantage of linguistic similarities or giving the resident a sense of security through a friendly tone of voice [33–35, 37, 38, 42, 43, 45, 46, 49, 50, 52]. Bilingual health professionals who share the resident’s preferred language naturally have an advantage in meeting the linguistic and cultural needs of CALD residents [55] and some of these spend one-on-one time with the resident to form a relationship [38]. They are also often given the role of interpreter or cultural broker [38, 40, 43–45, 49, 50, 52], informing their colleagues of the resident’s needs. There is also an instance of the bilingual health professional bringing home-cooked ethnic meals to the resident [56].

The CALD resident’s immediate family also holds an important supporting role after the resident has moved into a care home. Not only do they often promote culturally appropriate care through helping in arranging culturally significant activities or providing ethnic meals [38, 43], they also act as interpreters and facilitate communication between their loved one and responsible health professionals [37, 40, 42–44, 47, 50, 52]. Care homes also sometimes form collaborations with cultural or community organisations [40, 43, 44, 50] or individuals from outside of the care home setting to promote cultural inclusivity and facilitate communication. Professional interpreters and bilingual volunteers can sometimes be invited to interpret for the resident [35, 52]. Some studies also mention that bilingual volunteers help with the care of CALD residents by collecting life stories, helping health professionals with health documentation and providing ethnic meals, thus contributing to improving the quality of care for CALD residents [34, 35].

#### Attitudes (e4)

The attitudes of surrounding people are also an essential part of CALD residents’ care home environments. These attitudes may be based on these surrounding people’s own norms, customs and beliefs and may affect the way they treat others. The results of this review stress the important roles of health professionals and some of these are described as showing supportive attitudes through cultural awareness [35, 45, 50, 51] and through embracing cultural differences [34] in the care home. These professionals understand the importance of creating opportunities for linguistic stimulation [38, 42] for minority residents and acknowledge how ethnic food [38, 51, 56] contributes to making CALD residents feel like home. Other health professionals, however, are described as displaying negative attitudes. Examples of this can be seen when some health professionals show a lack of respect toward the resident and their family members [47], underestimate CALD residents’ cognitive abilities because of language barriers [57] or state that they do not see why the absence of traditional ethnic food is a problem [56].

An understanding of each resident’s cultural norms is important to make them feel comfortable and respected. Norms related to gender roles is one such example. Studies describe how female CALD residents feel upset when male health professionals enter their room without permission or try to be involved in their hygiene routines [43]. Women of some cultures also have a need to be nurturing, and involving these in activities such as cooking or tasting the food helps them keep their role as the matriarch [43]. To understand cultural norms related to showing intergenerational or professional respect is also important [58]. One study describes how a CALD resident felt like he lost his personal identity and sense of self-worth when health professionals repeatedly used the wrong form of address when speaking to him [57].

#### Services, systems and policies (e5)

Other than the use of assistive technologies to overcome communicative barriers, some studies also contained examples of specific interventions with the purpose of creating an inclusive care environment for CALD residents. One example of this is a Montessori-inspired activity programme through which care professionals were able to engage residents in stimulating activities across linguistic barriers. Common activities could be looking at and sorting pictures, arranging flowers or listening and singing along to favourite music [59]. These activities were shown to decrease agitated behaviours among CALD residents with dementia who lacked fluency in English. Another example is a participatory arts programme, through which health professionals could create a failure-free environment for residents to playfully investigate linguistic and cultural differences and consequently place all those involved in the activity on an equal footing [53]. One care home also invited medical clowns who offered individually tailored entertainment after having read each resident’s life story [41]. The entertainment could contain elements of culturally significant music or dance, which provoked reminiscence among CALD residents and often entertained across cultural and linguistic barriers.

To be able to meet cultural and linguistic needs of CALD residents cultural care competence is crucial. Several studies mention that health professionals and leadership in care homes with CALD residents should be offered training in cultural or linguistic competence [40, 43, 44, 50], or strategies for alternative ways of communicating across linguistic barriers [43]. A professional understanding of cultural differences and needs could enable a higher quality of care.

**Table 3:** Overview of main findings related to environmental factors

ICF code	Category	Variation within code	Occurrence *n* = 27
e110	Products and substances for personal consumption	This code gathers meaningful concepts related to physical elements of the CALD resident’s environment that are used for personal consumption. This may include ethno-specific foods and drinks.	16 (59,3%)
e310	Immediate family	This code gathers meaningful concepts related to the role of immediate family members of CALD residents. This may include both their social importance to the resident and their importance to the facility in helping to promote culturally appropriate care.	12 (44,4%)
e355	Health professionals	This code gathers meaningful concepts related to the role of health professionals in the care home. This may include both the facilitating roles of health professionals who share a language with a CALD resident, as well as information on communicative strategies of health professionals who do not share a verbal language with the resident. The category also lists less constructive care strategies of health professionals.	20 (74,1%)
e450	Individual attitudes of health professionals	This code gathers meaningful concepts related to positive and negative attitudes among health professionals that may affect the CALD residents’ quality of care. This may include acknowledgements of the importance of cultural awareness as well as health professionals showing lack of respect.	13 (48,1%)
e580	Health services, systems and policies	This code gathers meaningful concepts related to types of care services, care programmes and routines used in the care homes. This category also includes indications of a need for staff training within various elements relevant to culturally appropriate care.	17 (63,0%)

## Discussion

This integrative review underlines the complexity of using environments to meet the culture-specific needs of CALD care home residents with dementia. The main findings highlight the importance of bilingual staff, culturally relevant activities and inclusive environments in enhancing communication, building interpersonal relationships and reducing frustration. While culturally significant products and technologies foster a sense of belonging and engagement among residents, training care home staff in cultural competence and involving families and cultural communities is essential for creating inclusive, respectful care. If appropriate cultural care is not delivered, CALD care home residents with dementia risk facing disadvantages and inequities in mainstream care homes [17, 60]. Several interesting discussion areas related to how this can be prevented have emerged from this review. Three such areas are the importance of creating a sense of home, preventing social isolation among CALD residents and the importance of a progressive organisational environment.

### Creating a sense of home

Transitioning into a care home can be accompanied by stress and lead to disruptive behaviours or depression among new residents [39]. The physical living environment is one factor which affects a person’s experiences of transitioning. Familiar environmental elements, such as personal possessions, familiar food and access to recognisable media, have been shown to reinforce the resident’s sense of identity and connection to their past [61]. Elements of the physical environment in care homes can be used to evoke recognition and to give residents a sense of home [62, 63]. Examples of this could be the taste and smell of ethnic food or the use of furnishings which are recognisable to the resident. If these elements are placed in the common room, they may even stimulate social engagement between residents. The results of this integrative review are in line with these findings and stress the importance of ethnic food but also highlight that many care homes found it challenging to provide such food regularly due to lack of cultural knowledge or resources. Instead, ethnic food was often provided by people from outside of the care home organisation, such as family members, volunteers or members of cultural or religious communities. Perhaps stronger and more systematic cooperation between care homes and cultural communities may enable more CALD residents to enjoy familiar food and increase the cultural awareness of care home staff. Familiar furnishings were also mentioned, but these were often kept in the residents’ own private rooms instead of being displayed in the common room. The results of this review therefore highlight the importance of community and how limiting culturally familiar furnishings to the residents’ private rooms are a missed opportunity to let these physical elements of the care home environment contribute to social stimulation.

### Preventing social isolation among CALD residents

Maintaining social activity is very important for the cognitive health of older adults. Perceived loneliness has been shown to increase the risk of developing some types of dementia [64]. There are indications that healthy lifestyle habits, such as being physically and socially active, should have a positive impact on the progression rate of dementia [65]. Furthermore, maintaining meaningful social relationships seems to have a positive effect on residents’ experiences of transitioning into a care home [61]. CALD residents may, compared with residents from the majority population, find it more difficult to engage in meaningful social activities in a mainstream care home due to cultural differences and linguistic barriers. Communication barriers may also prevent CALD residents from exercising their autonomy as they might face greater difficulties in expressing their wishes. This could lead to inequality in care delivery between the two groups. It is therefore of great importance to find strategies to secure equal opportunities for CALD residents to engage in social and physical activities.

One strategy for decreasing the risk of social isolation among CALD residents which was identified through this review was to look beyond the four walls of the care home and build a larger care community. The advantage of inviting residents’ families, bilingual volunteers and cultural or religious organisations to help meet culture-specific needs of CALD residents was most prominent [48]. These assistive groups were shown to function as cultural and linguistic brokers between residents and health professionals, help with health documentation and prevent social isolation among CALD residents. Furthermore, they also contributed to an increase in culturally relevant activities for CALD residents, that sometimes succeeded in engaging other residents and health professionals too. Such activities could contribute to creating an inclusive environment for CALD residents with dementia in care homes.

It is, however, not enough to rely on relatives and volunteers from outside of the care home to enhance the social environment of CALD residents. It is also important to find alternative strategies within the care homes themselves for decreasing the risk of social isolation among CALD residents. Our findings may inspire the further development and use of advanced, user-friendly linguistic solutions, such as AI-powered translation devices tailored for dementia care. For example, a recent study described the development of a conversational AI assistant that supported caregivers of individuals with dementia by dynamically adapting its vocabulary, sentence structure, and explanatory depth to match each caregiver’s literacy level and the resident’s communicative abilities [66]. AI has not only the potential to translate between languages, but also to enhance communication and personalise care.

### The importance of a progressive organisational environment

CALD residents make up an increasing percentage of older adults with dementia who need to move into care homes. It is necessary for these institutions to be progressive and to adapt their operations to this reality. While family and community members are an integral part of a CALD resident’s social and cultural environment in the care home, it is the responsibility of the management to ensure person-centred care [67, 68]. Each resident in the care home should receive care that enables them to achieve their full health potential. This review has highlighted some limitations of mainstream care homes in offering their CALD residents a culturally appropriate and familiar care environment. To take part of the benefits of cooperating with family and community members, the care homes should be active in organising cultural events which encourage community participation. At the same time, it is important that the responsibility remains within the care home and is not placed on family members, as research has shown that involvement in the care of dementia patients can cause distress among family members, as they feel uncertain about their loved one’s care needs and abilities to connect [69]. The results of this review indicate that there was a desire among health professionals to learn more about culturally competent care. Given the increase in global migration and growth of multicultural societies it should be a priority of people in leadership positions to make sure that their health professionals acquire this cultural competence and receive means for communicating across language barriers. Such skills and aids are likely to help health professionals in their strive to offer all residents equitable care.

### The use of environments and person-centred care

Persons with dementia should be treated with person-centred care [70]. According to a person-centred approach each resident in a care home should be seen as unique with their own set of needs and preferences [71]. A person-centred care giver should therefore try to form a partnership with each individual resident and develop a care-plan in collaboration with them, taking that specific resident’s expressed experiences and preferences into account [26]. It may therefore be questioned how any common conclusions could be drawn regarding using environmental elements to meet culture-specific needs of such a diverse group of people, whose only common trait may be their CALD background. In fact, only four articles (15%) included in this review, mentioned person-centred care at all. A greater understanding of how culture-specific needs of CALD residents could be met using environments may, however, be eye-opening to available options and therefore facilitate the future process of tailoring interventions to each unique person.

### Strengths and limitations

Despite a systematic and comprehensive search strategy for scientific publications, there is the potential that not all relevant literature was identified. No grey literature was included or searched for. We did, however, include publications in all languages in our search in order to not exclude important publications based on language alone. There were only a few non-English publications among the set of articles, but they were all excluded at the title and abstract screening phase as none of them met the inclusion criteria.

The process of article inclusion and data extraction were performed by two authors to increase the reliability of both processes. Similarly, two authors performed quality appraisal in order to increase the reliability of appraisal. The ICF based data analysis was mainly performed by the first author after having received instructions from the second author, who is an expert in using ICF in different groups of functional impairments. A randomized 50% sample of the data analysis was also controlled by the second author at completion.

#### The use of the ICF framework for data analysis

The ICF framework is a globally agreed framework for classifying and understanding health-related determinants [22]. In line with recent ICF based reviews in dementia and elder care for the general population [23, 72, 73], the ICF seemed appropriate in unpacking the complexity of dementia care for CALD residents and providing a common language to facilitate communication among different healthcare professions. The framework contributes with a well-developed and thought-out structure which distinguishes between the resident’s perspectives and external environmental factors. As such it is well-adjusted for research within occupational therapeutic research on supportive environments. The ICF framework also allows for codes to be accompanied by qualifiers, enabling measurable comparisons within health-related determinants. These were, however, not applicable in the process of reviewing previous research as the magnitude of the supporting or opposing factors could not always be determined in the included studies. Thus, a direct comparison of the use of environmental factors to meet culture-specific needs of CALD residents could not be made between the included studies.

A limitation of using the ICF in the analysis process could be that some categories seem to overlap, occasionally making it difficult to choose between two similar codes. Some descriptions of codes are also difficult to interpret, making it hard to determine if they really fit the linking unit well. Furthermore, there is also an imbalance in the granularity of codes, with some being very detailed and others being less so. While some data can therefore be specified in detail through the selected codes, others need to be coded in a less detailed manner.

## Conclusion

Relatively few studies (*n* = 27) were found that could shed light on how environments in care homes could be used to meet culture-specific needs of CALD residents with dementia. These studies generally did not have the aim to look at this issue directly but rather contributed with different perspectives that illuminated CALD residents’ experiences of living in care homes while having dementia. From these findings, environmental elements of importance could then be extracted. The results include the perspectives of both CALD residents and their family members, health professionals, management and observations by researchers and they point in the same direction. An unequal opportunity for CALD residents with dementia to feel at home and maintain a social context in the care home is evident and there is a need for professional development in culturally competent care among health professionals. The findings emphasize the importance of cultural awareness among health professionals and the importance of creating a greater care community to meet the social and cultural needs of CALD residents. Such a community could help turn the care home environment into a home for CALD residents. More research is needed on how to best make such cooperation possible.

## Supplementary Information

Below is the link to the electronic supplementary material.

Supplementary Material 1

Supplementary Material 2

Supplementary Material 3

## Data Availability

The datasets used and/or analysed during the current study are available from the corresponding author upon reasonable request.
