# Supplementary material for: The use of care home environments to meet culture-specific needs of culturally and linguistically diverse residents with dementia: an integrative review using the ICF framework
Source: Int J Equity Health. 2026 Jan 16;25:15. doi: 10.1186/s12939-025-02748-0 (PMC12817771; doi:10.1186/s12939-025-02748-0)
Supplement: Supplementary file 1 — Supplementary Material 1 [file 12939_2025_2748_MOESM1_ESM.docx]

**Total:** 4311 studies **Duplicates identified by Covidence:** 1904 **Total to screen:** 2243

**CINAHL with Full Text via EBSCO, 2024-12-18**

| Search terms | | | Results |
| --- | --- | --- | --- |
| **Nursing Homes** | | | |
|  | 1. | MH "Nursing Homes" OR MH "Nursing Home Residents" OR MH "Residential Facilities" OR MH "Residential Care" OR MH "Assisted Living" OR MH "Housing for Older Persons" | 51,495 |
|  | 2. | TI ( "long term care" OR "longterm care" OR "sheltered accommodation*" OR "sheltered housing" OR "care home*" OR "residential home*" OR "nursing home*" OR "residential age* care" OR "residential facilit*" OR "senior housing" OR "home* for the aged" OR "housing for old*" OR "housing for the elderly" OR “extra care housing” OR “dementia village*” ) OR AB ( "long term care" OR "longterm care" OR "sheltered accommodation*" OR "sheltered housing" OR "care home*" OR "residential home*" OR "nursing home*" OR "residential age* care" OR "residential facilit*" OR "senior housing" OR "home* for the aged" OR "housing for old*" OR "housing for the elderly" OR “extra care housing” OR “dementia village*” ) | 49,539 |
|  | 3. | 1 OR 2 | 74,704 |
| **Cognition disorder** | | | |
|  | 4. | MH "Dementia+" OR MH "Dementia Patients" OR MH "Memory Disorders" OR MH "Cognition Disorders" | 117,633 |
|  | 5. | TI (dementia OR alzheimer* OR "memory disorder*" OR "memory loss" OR "memory impair*" OR "memory deficit*" OR "memory dysfunction*" OR "cognition disorder*" OR “cognition impair*” OR "cognitive loss" OR "cognitive impair*" OR "cognitive deficit*" OR "cognitive dysfunction*" OR "cognitive decline*" OR "cognitive defect*" OR “cognitive disorder*”)) OR (AB (dementia OR alzheimer* OR "memory disorder*" OR "memory loss" OR "memory impair*" OR "memory deficit*" OR "memory dysfunction*" OR "cognition disorder*" OR “cognition impair*” OR "cognitive loss" OR "cognitive impair*" OR "cognitive deficit*" OR "cognitive dysfunction*" OR "cognitive decline*" OR "cognitive defect*" OR “cognitive disorder*”) | 126,877 |
|  | 6. | 4 OR 5 | 162,717 |
| **Culture** | | | |
|  | 7. | MH "Culture" OR MH "Cultural Diversity" OR MH "Cultural Competence" OR MH "Cultural Sensitivity" OR MH "Transcultural Care" OR MH "Language OR MH "Multilingualism" OR MH "Linguistics" OR MH "Ethnic Groups" OR MH "Minority Groups" OR MH "Immigrants" | 68,020 |
|  | 8. | TI (cultur* OR multicultur* OR language* OR linguistic* OR ethni* OR multilingual* OR bilingual* OR transcultural* OR emigra* OR immigra* OR migra*)) OR (AB (cultur* OR multicultur* OR language* OR linguistic* OR ethni* OR multilingual* OR bilingual* OR transcultural* OR emigra* OR immigra* OR migra*) | 415,730 |
|  | 9. | 7 OR 8 | 441,488 |
| **Combined sets/Limits** | | | |
|  | 10. | 3 AND 6 AND 9 | 784 |
|  | 11. | Publication Year: 2013-2024 Peer Reviewed | 503 |

**MEDLINE via EBSCO 2024-12-18**

| Search terms | | | Results |
| --- | --- | --- | --- |
| **Nursing Homes** | | | |
|  | 1. | MH "Nursing Homes" OR MH "Residential Facilities" OR MH "Assisted Living Facilities" OR MH "Homes for the Aged" OR MH "Housing for the Elderly" OR MH "Long-Term Care" | 77,282 |
|  | 2. | TI ( "long term care" OR "longterm care" OR "sheltered accommodation*" OR "sheltered housing" OR "care home*" OR "residential home*" OR "nursing home*" OR "residential age* care" OR "residential facilit*" OR "senior housing" OR "home* for the aged" OR "housing for old*" OR "housing for the elderly" OR “extra care housing” OR “dementia village*” ) OR AB ( "long term care" OR "longterm care" OR "sheltered accommodation*" OR "sheltered housing" OR "care home*" OR "residential home*" OR "nursing home*" OR "residential age* care" OR "residential facilit*" OR "senior housing" OR "home* for the aged" OR "housing for old*" OR "housing for the elderly" OR “extra care housing” OR “dementia village*” ) | 69,333 |
|  | 3. | 1 OR 2 | 106,415 |
| **Cognition disorder** | | | |
|  | 4. | MH "Dementia+" OR MH "Memory Disorders" OR MH "Cognition Disorders+" | 320,794 |
|  | 5. | TI (dementia OR alzheimer* OR "memory disorder*" OR "memory loss" OR "memory impair*" OR "memory deficit*" OR "memory dysfunction*" OR "cognition disorder*" OR “cognition impair*” OR "cognitive loss" OR "cognitive impair*" OR "cognitive deficit*" OR "cognitive dysfunction*" OR "cognitive decline*" OR "cognitive defect*" OR “cognitive disorder*”)) OR (AB (dementia OR alzheimer* OR "memory disorder*" OR "memory loss" OR "memory impair*" OR "memory deficit*" OR "memory dysfunction*" OR "cognition disorder*" OR “cognition impair*” OR "cognitive loss" OR "cognitive impair*" OR "cognitive deficit*" OR "cognitive dysfunction*" OR "cognitive decline*" OR "cognitive defect*" OR “cognitive disorder*”) | 420,890 |
|  | 6. | 4 OR 5 | 503,081 |
| **Culture** | | | |
|  | 7. | MH "Culture" OR MH "Cultural Diversity" OR MH "Cultural Competency" OR MH "Culturally Competent Care" OR MH "Language" OR MH "Multilingualism" OR MH "Linguistics" OR MH "Ethnicity" OR MH "Minority Groups" OR MH "Emigrants and Immigrants" OR MH "Transients and Migrants" | 227,333 |
|  | 8. | TI (cultur* OR multicultur* OR language* OR linguistic* OR ethni* OR multilingual* OR bilingual* OR transcultural* OR emigra* OR immigra* OR migra*)) OR (AB (cultur* OR multicultur* OR language* OR linguistic* OR ethni* OR multilingual* OR bilingual* OR transcultural* OR emigra* OR immigra* OR migra*) | 2,233,206 |
|  | 9. | 7 OR 8 | 2,321,752 |
| **Combined sets/Limits** | | | |
|  | 10. | 3 AND 6 AND 9 | 1,016 |
|  | 11. | Publication Year: 2013-2024 Peer Reviewed | 667 |

**APA PsycInfo via EBSCO 2024-12-18**

| Search terms | | | Results |
| --- | --- | --- | --- |
| **Nursing Homes** | | | |
|  | 1. | DE "Nursing Homes" OR DE "Nursing Home Residents" OR DE "Residential Care Institutions" OR DE "Assisted Living" OR DE "Long Term Care" | 29,652 |
|  | 2. | TI ( "long term care" OR "longterm care" OR "sheltered accommodation*" OR "sheltered housing" OR "care home*" OR "residential home*" OR "nursing home*" OR "residential age* care" OR "residential facilit*" OR "senior housing" OR "home* for the aged" OR "housing for old*" OR "housing for the elderly" OR “extra care housing” OR “dementia village*” ) OR AB ( "long term care" OR "longterm care" OR "sheltered accommodation*" OR "sheltered housing" OR "care home*" OR "residential home*" OR "nursing home*" OR "residential age* care" OR "residential facilit*" OR "senior housing" OR "home* for the aged" OR "housing for old*" OR "housing for the elderly" OR “extra care housing” OR “dementia village*” ) | 25,509 |
|  | 3. | 1 OR 2 | 39,082 |
| **Cognition disorder** | | | |
|  | 4. | DE "Dementia" OR DE "Alzheimer's Disease" OR DE "Memory Disorders" OR DE "Cognitive Impairment" | 141,338 |
|  | 5. | TI (dementia OR alzheimer* OR "memory disorder*" OR "memory loss" OR "memory impair*" OR "memory deficit*" OR "memory dysfunction*" OR "cognition disorder*" OR “cognition impair*” OR "cognitive loss" OR "cognitive impair*" OR "cognitive deficit*" OR "cognitive dysfunction*" OR "cognitive decline*" OR "cognitive defect*" OR “cognitive disorder*”)) OR (AB (dementia OR alzheimer* OR "memory disorder*" OR "memory loss" OR "memory impair*" OR "memory deficit*" OR "memory dysfunction*" OR "cognition disorder*" OR “cognition impair*” OR "cognitive loss" OR "cognitive impair*" OR "cognitive deficit*" OR "cognitive dysfunction*" OR "cognitive decline*" OR "cognitive defect*" OR “cognitive disorder*”) | 186,483 |
|  | 6. | 4 OR 5 | 203,345 |
| **Culture** | | | |
|  | 7. | DE "Culture (Anthropological)" OR DE "Cultural Diversity" OR DE "Cultural Sensitivity" OR DE "Cultural Competence" OR DE "Multiculturalism" OR DE "Ethnic Identity" OR DE "Ethnic Diversity" OR DE "Racial and Ethnic Groups" OR DE "Minority Groups" OR DE "Immigration" OR DE "Language" OR DE "Foreign Languages" OR DE "Multilingualism" OR DE "Bilingualism" OR DE "Linguistics" OR DE "Cross Cultural Communication" | 211,931 |
|  | 8. | TI (cultur* OR multicultur* OR language* OR linguistic* OR ethni* OR multilingual* OR bilingual* OR transcultural* OR emigra* OR immigra* OR migra*)) OR (AB (cultur* OR multicultur* OR language* OR linguistic* OR ethni* OR multilingual* OR bilingual* OR transcultural* OR emigra* OR immigra* OR migra*) | 740,416 |
|  | 9. | 7 OR 8 | 773,095 |
| **Combined sets/Limits** | | | |
|  | 10. | 3 AND 6 AND 9 | 701 |
|  | 11. | Publication Year: 2013-2024 Peer Reviewed | 352 |

**ASSIA (Applied Social Sciences Index and Abstracts) via ProQuest 2024-12-18**

| Search terms | | | Results |
| --- | --- | --- | --- |
| **Nursing Homes** | | | |
|  | 1. | MAINSUBJECT.EXACT("Nursing homes" OR "Long term health care" OR "Assisted living facilities" OR "Residential care") | 13,747 |
|  | 2. | title("long term care" OR "longterm care" OR "sheltered accommodation*" OR "sheltered housing" OR "care home*" OR "residential home*" OR "nursing home*" OR "residential age* care" OR "residential facilit*" OR "senior housing" OR "home* for the aged" OR "housing for old*" OR "housing for the elderly" OR “extra care housing” OR “dementia village*” ) OR abstract("long term care" OR "longterm care" OR "sheltered accommodation*" OR "sheltered housing" OR "care home*" OR "residential home*" OR "nursing home*" OR "residential age* care" OR "residential facilit*" OR "senior housing" OR "home* for the aged" OR "housing for old*" OR "housing for the elderly" OR “extra care housing” OR “dementia village*” ) | 14,495 |
|  | 3. | 1 OR 2 | 19,012 |
| **Cognition disorder** | | | |
|  | 4. | MAINSUBJECT.EXACT("Dementia" OR "Alzheimers disease" OR "Memory disorders" OR "Cognitive impairment") | 17,941 |
|  | 5. | title(dementia OR alzheimer* OR "memory disorder*" OR "memory loss" OR "memory impair*" OR "memory deficit*" OR "memory dysfunction*" OR "cognition disorder*" OR “cognition impair*” OR "cognitive loss" OR "cognitive impair*" OR "cognitive deficit*" OR "cognitive dysfunction*" OR "cognitive decline*" OR "cognitive defect*" OR “cognitive disorder*”) OR abstract(dementia OR alzheimer* OR "memory disorder*" OR "memory loss" OR "memory impair*" OR "memory deficit*" OR "memory dysfunction*" OR "cognition disorder*" OR “cognition impair*” OR "cognitive loss" OR "cognitive impair*" OR "cognitive deficit*" OR "cognitive dysfunction*" OR "cognitive decline*" OR "cognitive defect*" OR “cognitive disorder*”) | 24,700 |
|  | 6. | 4 OR 5 | 27,075 |
| **Culture** | | | |
|  | 7. | MAINSUBJECT.EXACT("Culture" OR "Cultural tradition" OR "Multiculturalism & pluralism" OR "Cultural competence" OR "Cultural identity" OR "Cultural sensitivity" OR "Minority groups" OR "Ethnic groups" OR "Ethnicity " OR "Immigration" OR "Migrants" OR "Language" OR "Multilingualism" OR "Bilingualism" OR "Linguistics") | 54,922 |
|  | 8. | title(cultur* OR multicultur* OR language* OR linguistic* OR ethni* OR multilingual* OR bilingual* OR transcultural* OR emigra* OR immigra* OR migra*) OR abstract(cultur* OR multicultur* OR language* OR linguistic* OR ethni* OR multilingual* OR bilingual* OR transcultural* OR emigra* OR immigra* OR migra*) | 166,666 |
|  | 9. | 7 OR 8 | 177,186 |
| **Combined sets/Limits** | | | |
|  | 10. | 3 AND 6 AND 9 | 303 |
|  | 11. | Publication Year: 2013-2024 Peer Reviewed | 177 |

**Web of Science 2024-12-18**

| Search terms | | | Results |
| --- | --- | --- | --- |
| **Nursing Homes** | | | |
|  | 1. | TS=( "long term care" OR "longterm care" OR "sheltered accommodation*" OR "sheltered housing" OR "care home*" OR "residential home*" OR "nursing home*" OR "residential age* care" OR "residential facilit*" OR "senior housing" OR "home* for the aged" OR "housing for old*" OR "housing for the elderly" OR "extra care housing" OR "dementia village*" ) | 81,438 |
| **Cognition disorder** | | | |
|  | 2. | TS=( "dementia" OR alzheimer* OR "memory disorder*" OR "memory loss" OR "memory impair*" OR "memory deficit*" OR "memory dysfunction*" OR "cognition disorder*" OR "cognition impair*" OR "cognitive loss" OR "cognitive impair*" OR "cognitive deficit*" OR "cognitive dysfunction*" OR "cognitive decline*" OR "cognitive defect*" OR "cognitive disorder*") | 577,629 |
| **Culture** | | | |
|  | 3. | TS=( cultur* OR multicultur* OR language* OR linguistic* OR ethni* OR multilingual* OR bilingual* OR transcultural* OR emigra* OR immigra* OR migra* ) | 4,462,464 |
| **Combined sets/Limits** | | | |
|  | 4. | 1 AND 2 AND 3 | 1,593 |
|  | 5. | Publication Year: 2013-2025 | 1201 |

**Scopus 2024-12-18**

| Search terms | | | Results |
| --- | --- | --- | --- |
| **Nursing Homes** | | | |
|  | 1. | TITLE-ABS-KEY ( "long term care" OR "longterm care" OR "sheltered accommodation*" OR "sheltered housing" OR "care home*" OR "residential home*" OR "nursing home*" OR "residential age* care" OR "residential facilit*" OR "senior housing" OR "home* for the aged" OR "housing for old*" OR "housing for the elderly" OR "extra care housing" OR "dementia village*" ) | 254,488 |
| **Cognition disorder** | | | |
|  | 2. | TITLE-ABS-KEY (dementia OR alzheimer* OR "memory disorder*" OR "memory loss" OR "memory impair*" OR "memory deficit*" OR "memory dysfunction*" OR "cognition disorder*" OR "cognition impair*" OR "cognitive loss" OR "cognitive impair*" OR "cognitive deficit*" OR "cognitive dysfunction*" OR "cognitive decline*" OR "cognitive defect*" OR "cognitive disorder*") | 702,751 |
| **Culture** | | | |
|  | 3. | TITLE-ABS-KEY ( cultur* OR multicultur* OR language* OR linguistic* OR ethni* OR multilingual* OR bilingual* OR transcultural* OR emigra* OR immigra* OR migra* ) | 6,949,579 |
| **Combined sets/Limits** | | | |
|  | 4. | 1 AND 2 AND 3 | 2,200 |
|  | 5. | Publication Year: 2013-2025 Source Type: Journal | 1,411 |
