# Supplementary material for: The use of care home environments to meet culture-specific needs of culturally and linguistically diverse residents with dementia: an integrative review using the ICF framework
Source: Int J Equity Health. 2026 Jan 16;25:15. doi: 10.1186/s12939-025-02748-0 (PMC12817771; doi:10.1186/s12939-025-02748-0)
Supplement: Supplementary file 2 — Supplementary Material 2 [file 12939_2025_2748_MOESM2_ESM.docx]

Appendix 2: Quality appraisal of the included studies using the Mixed Methods Appraisal Tool (MMAT)

| **Qualitative studies** | | | | | | | | | | | | | | | | |  |
| --- | --- | --- | --- | --- | --- | --- | --- | --- | --- | --- | --- | --- | --- | --- | --- | --- | --- |
| Study | | Are there clear research questions? | | Do the collected data allow to address the research questions? | | Is the qualitative approach appropriate to answer the research questions? | | Are the qualitative data collection methods adequate to address the research question? | | Are the findings adequately derived from the data? | | Is the interpretation of results sufficiently substantiated by data? | | Is there coherence between data sources, collection, analysis and interpretation? | Overall quality description by assessors |  |  |
| Chin et al (2019) | | Y | | Y | | Y | | Y | | Y | | Y | | Y | Met key criteria using an ethnographic approach and thematic analysis, coherent findings |  |  |
| du Toit et al (2020) | | Y | | Y | | Y | | Y | | Y | | Y | | Y | While the adapted Delphi method is appropriate for gathering expert consensus, the low response rate and lack of sustained participation in later rounds limited robustness of the data |  |  |
| Hanssen (2013) | | Y | | Y | | Y | | Y | | Y | | Y | | Y | Appropriate hermeneutic thematic analysis demonstrated the impact of cultural background on dementia care, though small sample size |  |  |
| Hanssen & Kuven (2016) | | Y | | Y | | Y | | Y | | Y | | Y | | Y | Appropriate hermeneutic content-focused analysis captured lived experiences, emotions, and cultural meanings associated with food |  |  |
| Hung et al (2023) | | Y | | Y | | Y | | Y | | Y | | Y | | Y | Focus groups captured shared experiences and professional insights, presented well-supported findings |  |  |
| Inoue et al (2021) | | Y | | Y | | Y | | Y | | Y | | Y | | Y | semi-structured interviews and thematic analysis to identify benefits, though its reliance on staff interpretations without patient observations |  |  |
| Jansson (2014) | | Y | | Y | | Y | | Y | | Y | | Y | | Y | Used ethnographic observations, audio/video recordings, and interviews to analyze real-life caregiving interactions. Linguistic ethnography and conversation analysis, though a single case study |  |  |
| Juul et al (2019) | | Y | | Y | | Y | | Y | | C | | C | | Y | Ethnographic fieldwork, participant observations, interviews, and video recordings were appropriate. Can’t tell if results were sufficiently substantiated by data and the coherence between data sources, collection, analysis and interpretation |  |  |
| Kiwi (2019) | | Y | | Y | | Y | | Y | | Y | | Y | | Y | Appropriate semi-structured interviews, ethnographic fieldwork, and qualitative content analysis to reveal how cultural expectations shape caregiving decisions |  |  |
| Kiwi (2023) | | Y | | Y | | Y | | Y | | Y | | Y | | Y | Appropriate ethnographic fieldwork, dementia-friendly interviews, and thematic content analysis to capture identity, belonging, and adaptation |  |  |
| Koehn et al (2018) | | Y | | Y | | Y | | Y | | Y | | Y | | Y | Semi-structured interviews, observations, and family council analysis were appropriate. Thematic analysis and intersectionality were adequate |  |  |
| Rämgård et al (2016) | | Y | | Y | | Y | | Y | | Y | | Y | | Y | Appropriate use of ethnographic fieldwork and observational data to analyze interaction strategies |  |  |
| Rosendahl et al (2016) | | Y | | Y | | Y | | Y | | Y | | Y | | Y | Good semi-structured interviews and thematic analysis to examine linguistic barriers and cultural adaptation |  |  |
| Small et al (2015) | | Y | | Y | | Y | | Y | | Y | | Y | | Y | Rigorous video-recorded observations, transcription, and qualitative coding to analyze verbal and nonverbal behaviors. Using communication accommodation theory, it identifies strategies that enhance or hinder positive interaction |  |  |
| Söderman et al (2016) | | Y | | Y | | Y | | Y | | Y | | Y | | Y | Appropriate semi-structured interviews and thematic content analysis to examine communication barriers, language loss, and culturally adaptation |  |  |
| Strandroos & Antelius (2017) | | Y | | Y | | Y | | Y | | Y | | Y | | Y | Effective use of ethnographic fieldwork, video recordings, and caregiver interviews to analyze verbal and nonverbal strategies. Appropriate thematic analysis |  |  |
| Swinnen & de Medeiros (2018) | | Y | | Y | | Y | | Y | | Y | | Y | | Y | Appropriate use of ethnographic fieldwork, participant observations, audio recordings, and facilitator reflections to analyze how language play enhances engagement and communication. Linguistic and thematic analysis brought depth in the results |  |  |
| Wareing & Sethares (2021) | | Y | | Y | | Y | | Y | | Y | | Y | | Y | Focus group methodology and thematic content analysis were used appropriately to explore emotional, social, cultural, and institutional challenges |  |  |
| Xiao, LD et al (2023) | | Y | | Y | | Y | | Y | | Y | | Y | | Y | Appropriate use of focus groups and thematic analysis to examine communication barriers, cultural adaptation, and teamwork |  |  |
| Xiao, L et al (2023) | | Y | | Y | | Y | | Y | | Y | | Y | | Y | Appropriate use of resident and family interviews, focus groups, and thematic analysis to examine communication barriers, autonomy in care decisions, and social engagement |  |  |
| Yazdanpanah (2022) | | Y | | Y | | Y | | Y | | Y | | Y | | Y | Ethnographic fieldwork, interviews, and thematic analysis were used appropriately to explore mismatches between staff naming and resident preferences |  |  |
| **Quantitative Randomised Controlled Trials** | | | | | | | | | | | | | | | | | |
| Study | Are there clear research questions? | | Do the collected data allow to address the research questions? | | Is randomisation appropriately performed? | | Are the groups comparable at baseline? | | Are there complete outcome data? | | Are outcome assessors blinded to the intervention provided? | | Did the participants adhere to the assigned intervention? | | Overall quality description by assessors | | |
| van der Ploeg et al (2013) | Y | | Y | | Y | | Y | | Y | | N | | Y | | Randomized crossover trial design, structured measures, and statistical analysis showed good internal validity. The assessors were not blinded to the intervention | | |
| **Quantitative descriptive studies** | | | | | | | | | | | | | | | | | |
| Study | Are there clear research questions? | | Do the collected data allow to address the research questions? | | Is the sampling strategy relevant to address the research question? | | Is the sample representative of the target population? | | Are the measurements appropriate? | | Is the risk of nonresponse bias low? | | Is the statistical analysis appropriate to answer the research question? | | Overall quality description by assessors | | |
| Haesook et al (2014) | Y | | Y | | Y | | Y | | Y | | C | | Y | | Real-time observational coding and statistical analysis were used appropriately to examine interactions. Can’t tell if the risk of nonresponse bias low. | | |
| Runci et al (2014) | Y | | Y | | Y | | Y | | Y | | Y | | Y | | A large-scale postal survey and statistical analysis was used appropriately to assess resident and staff language diversity, language-specific services, and gaps in support | | |
| **Mixed Methods** | | | | | | | | | | | | | | | | | |
| Study | Are there clear research questions? | | Do the collected data allow to address the research questions? | | Is there an adequate rationale for using a mixed methods design to address the research question? | | Are the different components of the study effectively integrated to answer the research question? | | Are the outputs of the integration of qualitative and quantitative components adequately interpreted? | | Are divergences and inconsistencies between quantitative and qualitative results adequately addressed? | | Do the different components of the study adhere to the quality criteria of each tradition of the methods involved? | | Overall quality description by assessors | | |
| Cooper et al (2018) | Y | | Y | | Y | | Y | | Y | | Y | | Y | | A well-justified mixed-methods design integrating qualitative and quantitative components effectively, interprets findings cohesively, and adheres to methodological quality standards | | |
| du Toit & Buchanan (2018) | Y | | Y | | Y | | Y | | Y | | Y | | Y | | Effectively integrates qualitative and quantitative components, and adheres to methodological quality standards, though it does not explicitly address divergences between qualitative and quantitative findings​ | | |
| du Toit et al (2023) | Y | | Y | | Y | | Y | | Y | | Y | | Y | | Effectively used the Nominal Group Technique to collect qualitative data addressing those questions, justifies its mixed-methods design by capturing diverse perspectives from care staff, family members, and community-dwelling older adults, integrates study components cohesively, interprets qualitative findings adequately, does not explicitly address divergences between different data sources | | |

Y=Yes, N=No, C=Can’t tell
