# Supplementary material for: The use of care home environments to meet culture-specific needs of culturally and linguistically diverse residents with dementia: an integrative review using the ICF framework
Source: Int J Equity Health. 2026 Jan 16;25:15. doi: 10.1186/s12939-025-02748-0 (PMC12817771; doi:10.1186/s12939-025-02748-0)
Supplement: Supplementary file 3 — Supplementary Material 3 [file 12939_2025_2748_MOESM3_ESM.docx]

Appendix 3: Article matrix

| **Author(s) and year** | **Country** | **First language / ethnicity of CALD residents** | **Aim** | **Study design**  Method(s)  Study participants | **Key findings related to objectives of integrative review** |
| --- | --- | --- | --- | --- | --- |
| Chin et al. (2019) | Singapore | Cantonese  Hainanese  Hokkien | To explore the experiences of nursing staff communicating with Singaporean Chinese with dementia in Singapore | **Qualitative**  Ethnography; nonparticipant observations and semi-structured interviews with nursing staff (n = 19). | - Family members as interpreters - Language barriers between residents and health professionals (HP) - Problems in assisting CALD residents with self-care - HP use nonverbal communication - Some HP have taken language classes |
| Cooper et al. (2018) | United Kingdom | British/Irish (7%),  White other (42,3%),  Asian (15,5%),  Black (14,1%),  Mixed/other (21,1%) | As not speaking English as a first language may lead to increased difficulties in communication with staff and other residents, we (1) tested our primary hypotheses that care home residents with dementia speaking English as a second language experience more agitation and overall neuropsychiatric symptoms, and (2) explored qualitatively how staff consider that residents' language, ethnicity, and culture might impact on how they manage agitation | **Mixed method**  The Cohen-Mansfield Agitation Inventory, residents who did not speak English as first language (n = 69)  The DEMQOL proxy, staff (n = 70) and family carers (n = 49)  Clinical Dementia Rating  The Neuropsychiatric Inventory, residents who did not speak Eng as first language (n = 69)  Interviews with staff (n = 25) | - Bilingual HP learn about CALD residents’ preferences - The importance of ethnic food - Insecurities among HP regarding CALD residents’ religious needs - Language barriers between CALD residents and HP - Opportunities to speak own language calms CALD residents down - HP use nonverbal communication - Lack of routines in care home on how to share information about cultural and social backgrounds of residents among HP. |
| du Toit & Buchanan (2018) | South Africa | South Africa has 11 official languages,  residents with European ancestry | To identify best-practice scenarios for supporting older adults with moderate to advanced dementia from culturally and linguistically diverse backgrounds who lived in care facilities | **Mixed method**  Preworkshop questionnaire  Appreciative-inquiry workshop (n = 5 from each of these groups: nursing aides, volunteers, relatives of residents with dementia, residents without dementia)  Adapted Delphi technique | - The importance of socialising with family - Socialising across cultural barriers through activities that involve music, dance and eating - The importance of cultural awareness among HP - Creating opportunities to speak own language and share religious practice with others of same faith - Importance of bilingual volunteers in finding life stories - Family members teach HP common words |
| du Toit et al. (2023) | Australia | Indian | To explore (1) the effectiveness of current culturally appropriate dementia care practices, and (2) weaknesses and barriers for providing culturally  appropriate dementia care | **Mixed method**  Nominal group technique involving care staff (n = 8), family of residents (n = 8), community-dwelling older adults (n = 7) | - HP with training in culturally appropriate dementia care arranged cultural activities - Gender-related cultural norms, in relation to self-care and household activities - Culturally meaningful events, such as movies, games, cooking, music, family-time and opportunities to reminiscence about the past. - Family or cultural organisations help promote culturally appropriate care - Ethnic meals provided by meal-services or family members - Family members as cultural brokers - HP learn some key words and use language cards - Linguistic and cultural barriers - Ethno-specific aged care more successful in creating culturally recognisable environments where CALD residents feel a sense of belonging. - Cultural activities and celebrations including music, food, dance and prayer involving family and other residents - Bilingual HP as interpreter - Requested training in alternative means for communication and understanding of cultural and religious norms |
| du Toit et al. (2020) | Australia, New Zealand, South Africa, United Kingdom | Southern European linguistic and cultural background | To report on an international project that explored how residential care leadership understand meaningful engagement for residents with dementia from culturally and linguistically diverse (CALD) backgrounds | **Qualitative**  Adapted Delphi method applied on people in various leadership positions (facility manager, clinical manager, nurses, non-clinical manager, human resources officer) in care homes (n = 18) | - Training in culturally appropriate care for leadership staff - HP to communicate with help from family members or bilingual staff - Events to share and celebrate different cultures - Fostering greater insight into residents’ cultural and religious practice through staff education |
| Haesook et al. (2014) | United States of America | Korean | To explore the relationship between non-Korean nursing assistants’ communication style and behavioural symptoms of dementia in Korean-American nursing home residents with dementia | **Quantitative**  Observations of nursing assistants (n = 28) and Korean-American residents (n = 20) using the modified Agitated Behavior Rating Scale (mABRS) and social interaction coding schemas. | - Importance of culturally appropriate communication according to cultural norms |
| Hanssen (2013) | Norway | Sami | To gain knowledge about how the original culture may influence communication and interaction with institutionalised  patients with dementia and of what particular cultural aspects may come to the fore, exemplified by Sami patients. | **Qualitative**  Narrative interviews with family members (n = 8) and nursing staff (n = 9) experienced with dementia care. | - Negative implications of language barriers. - Importance of cultural elements to facilitate recognition. - Importance of cultural awareness among staff. - Recognition of sight, smell and taste encourages CALD resident to speak. |
| Hanssen & Kuven (2016) | Norway, South Africa | Sami,  Ethnic Norwegian,  South Africans of European decent | To learn about the meaning of traditional food to institutionalised patients with dementia | **Qualitative**  In-depth interviews with family members (n = 26) and nurses experienced in dementia care (n = 31). | - Recognition is important to make CALD residents feel safe and like they belong - Ethnic food can lead to reminiscence - Recognition of sight, smell and taste encourages CALD resident to speak. |
| Hung et al. (2023) | Canada | Diverse Asian background | To understand the perspectives of nurses and healthcare providers on the potential function and practice considerations of using TV videos for people with moderate to severe dementia | **Qualitative**  Five focus groups with nurses and healthcare providers (n = 23) in a long-term care home. | - HP connects with CALD residents by providing culturally and linguistically appropriate TV videos - Videos of familiar places encourage residents to converse - TV videos of cultural celebrations recall memories of the past |
| Inoue et al. (2021) | United States of America | Japanese | To explore the staff perceptions of a culturally responsive companion program provided to an older Japanese woman with advanced dementia in the long-term care setting to understand the potential benefits of such a program | **Qualitative**  Case study involving Japanese resident with dementia (n = 1). | - Volunteers who shared cultural and linguistic background with resident help HP with communication, culturally sensitive car and benefit CALD resident’s health - Volunteers decorate resident’s room according to cultural traditions and bring home-cooked meals - HP use nonverbal communication |
| Jansson (2014) | Sweden | Kurdish  Kurmanji | To demonstrate how immigrant care workers use their multilingualism as a resource for dealing with their tasks in encounters with a monolingual Kurdish-speaking resident in a Swedish-speaking dementia unit | **Qualitative**  Participant observation (n = 1), audio/video recordings and interviews with staff (n = 3). | - HP learn key phrases in resident’s language and use language mixing and body language. - Connect across language barriers through playful language - Bilingual HP as cultural and linguistic brokers |
| Juul et al. (2019) | Australia | Not specified | To investigates the potential for new technologies to enhance quality of life and facilitate meaningful engagement in physical and social activities among culturally and linguistically diverse residents and staff in care facilities | **Qualitative**  Case study with ethnographic fieldwork tools: participant observations (n = 18), targeted informal conversations with residents, staff and visitors, video ethnography, in-depth semi-structured interviews with residents (n = 15), family members (n = 10) and staff (n = 5). | - The Sitdance activity encourages socialisation across linguistic and cultural barriers, preventing social isolation - HP unsure if learning how to use the Sitdance intervention should be prioritised |
| Kiwi (2019) | Sweden | Persian  Azerbaijani  or a mix of languages | To explore the Iranian families and relatives’ attitudes towards culturally profiled nursing homes in Sweden and to explore what makes the family members’ attitudes differ despite the fact that it was their decision to move their older family members into these nursing homes | **Qualitative**  Semi-structured interviews with family members who had previously been informal caregivers (n = 29), observations and field notes. | - Language barriers in mainstream nursing homes create frustration among CALD residents - Culturally profiled nursing home provides culturally appropriate food and HP who can communicate in resident’s preferred language - HP in the culturally profiled nursing homes do not understand cultural codes due to generational differences |
| Kiwi (2023) | Sweden | Persian  Azerbaijani  or a mix of languages | To explore what Iranian participants with dementia experience while living in a culturally profiled residential home in Sweden | **Qualitative**  Interviews with individuals with dementia (n = 10), observations and field notes. | - Culturally profiled nursing home advertises a culturally sensitive physical and social environment with many elements from the residents’ birth country - Traditional and national celebrations give residents a feeling of home - Traditional prayer room rarely used for its intended purpose - Ethnic food is essential - Closeness to HP and other residents with shared language and background can contribute to positive social relationships |
| Koehn et al. (2018) | Canada | Cantonese | To form an in-depth understanding of how cultural-contextual factors and social locations influence the prevalence, structural features, role, function, and participation in Family Councils | **Qualitative**  Case study approach including participant observations and individual in-depth semi-structured interviews. | - Language barriers exclude CALD residents and their family members from family councils. - CALD residents and family members communicate with HP using body language and gestures - Care home tries to meet cultural needs through culturally appropriate boards games, TV programmes and visits to ethnic restaurants - CALD residents complain about food and lack of understanding of cultural norms concerning food - CALD TV available in common rooms, but often not used with mainstream residents present - CALD residents who meet residents of same background at care home, have a better social environment then before moving to the care home |
| Rämgård et al. (2016) | Sweden | Finnish  Bosnian  Greek  Spanish  Afghan  Iraqi  Iranian | To identify interaction strategies employed by medical clowns in culturally diverse dementia care settings | **Qualitative**  Observations of interactions between medical clowns and residents with dementia. | - Medical clowns (MC) learn each resident’s life story before starting their activities - MC use culturally recognisable elements of dance and music, creating culturally appropriate entertainment - MC activities sometimes engage across cultural and linguistic barriers, creating a sense of togetherness |
| Rosendahl et al. (2016) | Sweden | Finnish  Estonian  Hungarian  Ingrian | To explore and describe the experiences of family members and professional caregivers regarding the care provided to immigrants with dementia in group homes in Sweden | **Qualitative**  In-depth semi-structured interviews with professional caregivers (n = 9) and family members (n = 5) of people with dementia with diverse cultural backgrounds. | - Familiar music and TV programmes in native language - Relatives of CALD residents think they prefer traditional food from original country - HP use nonverbal communication - HP misinterpret resident’s inability to communicate in main language as a wish to be alone - Speaking one’s mother tongue provides linguistic stimulation and enables residents to socialise and form relationships - Family members work as interpreters |
| Runci et al. (2014) | Australia | Greek  Italian | To identify specific aspects of care that increased satisfaction of family members of Greek and Italian residents with dementia in mainstream or ethno-specific aged care in Australia | **Quantitative**  Questionnaire completed by interview with a total of 83 family members (41 with relatives in ethno-specific care and 42 in mainstream care). | - CALD residents in ethno-specific care homes interacted more with co-residents at a higher quality - Ethno-specific care home better able to meet linguistic and cultural needs - Satisfaction with food higher at ethno-specific care home - More culturally specific activities, such as celebrating festive days or entertainment in the resident’s language, requested |
| Small et al. (2015) | Canada | Chinese  Punjabi  Italian  Dutch  Polish  Filipino  Hindi | The goals of the present study were to 1) document the verbal and nonverbal behaviours used by staff and residents in diverse interactions, and 2) identify and account for behaviours that either promoted or detracted from positive communication by drawing on principles from ‘Communication Accommodation Theory’ | **Qualitative**  Video-recorded observations of staff (n = 27) and residents (n = 27) during routine activities. | - HP learn key phrases in resident’s language - Family members and bilingual HP as interpreters - Professional interpreters brought in as an exception - Nonverbal communication used between residents and HP |
| Söderman et al. (2016) | Sweden | Russian  Finnish | To explore and describe the nursing staff’s experiences of caring for non-Swedish speaking persons living with dementia in a Finnish speaking group home in relation to a Swedish speaking group home in Sweden | **Qualitative**  Semi-structured interviews (n = 27). | - Bilingual HP stress importance to continue speaking to CALD residents in their native language to form relationships and reminiscence - Language barriers resulted in   - CALD residents’ needs not being met   - CALD residents being perceived as sicker than they are   - Disease progression due to lack of stimulus - The use of nonverbal communication - CALD residents appreciated being served traditional food - Traditions and habits of the original culture important to CALD residents - Music in native language important, but kept in own room in mainstream care homes |
| Strandroos & Antelius (2017) | Sweden | Finnish  Hungarian  Polish  Kurdish  Arabic | To explore what (collaborative) communicative resources are used in interactions and to illuminate issues concerning meaning and self-making in relation to dementia and the sharing – or non-sharing – of common ground | **Qualitative**  Ethnographic participant observations, interviews with staff (n = 6) and video recordings. | - Language barriers prevent CALD residents from being social - Bilingual HP facilitate social interactions and relationships - HP use body language and other nonverbal communication to overcome language barriers |
| Swinnen & de Medeiros (2018) | Netherlands | Indonesian  Malaysian | To examine connections between language, identity, and cultural difference in the context of participatory arts in residential dementia care. Specifically, it looks at how language differences become instruments for the language play that characterizes the participatory arts programs, TimeSlips and the Alzheimer’s Poetry Project | **Qualitative**  Spoken-word workshops (n = 20) for people with dementia. Eight people on average attended each workshop. | - Participatory arts programmes facilitate socialising across language barriers, enabling CALD residents to form informal relationships with co-inhabitants |
| van der Ploeg et al. (2013) | Australia | Not specified | To test if personalized one-to-one interaction activities based on Montessori principles will improve agitation, affect, and engagement more than a relevant control condition | **Quantitative**  Randomized crossover trial in nine residential facilities (n = 44). | - Montessori activities allowed participation and activity across language barriers, resulting in a fall in agitated behaviours among participants |
| Wareing & Sethares (2021) | United States of America | Not specified | To elicit information from Certified Nursing Assistants (CNAs) about their views of the personality, social, institutional and cultural factors that affect the success or failure of elders’ adaptation to nursing home life | **Qualitative**  One focus group with Certified Nursing Assistants (n = 6). | - Conversing with others who spoke their language helped elders feel at home - CALD residents are paired with residents of similar cultural and linguistic backgrounds in the common rooms. - Recognizing holiday traditions and cultural activities, and involving residents in preparation for these events |
| Xiao, LD et al. (2023) | Australia | Not specified | To explore and compare staff perceived challenges and facilitators in supporting resident self-determination in ethno-specific and mainstream nursing  homes | **Qualitative**  Five focus groups with various direct care workers (n = 29) | - Bilingual and bicultural HP and family members support other HP in cross-cultural communication - Presence of language barriers - Lack of training - Cue cards, picture books and Google translator to communicate with CALD residents - Cooperations with community organisations to offer appropriate activities - Montessori training facilitates interactions with CALD residents - Care plans emphasised CALD residents’ culture-related care needs and preferences - Inability to provide room for religious activity - Understand the cultural importance of family |
| Xiao, L et al. (2023) | Australia | Italian  Greek | To compare factors affecting residents fulfilling self-determination in ethno-specific and mainstream nursing homes | **Qualitative**  Individual interviews of focus group with residents (n = 24) and family members (n = 5). | - Bilingual HP essential for CALD residents to meet care needs - Family members communicate residents’ needs and preferences - Importance of ethic food - Differences in mainstream and ethno-specific nursing homes in meeting religious needs - Ethno-specific care environments enable communication in preferred language and connecting with others in cultural and religious activities |
| Yazdanpanah (2022) | Sweden | Different dialects of Farsi  Arabic | To investigate empirically the role of address forms for residents and care-providing staff in multilingual residential settings | **Qualitative**  Interviews with residents (n = 5) and staff members (n = 21).  Observational and interactional data of residents (n = 5) and care-providing staff (n = 18). | - Mismatch between residents preferred and HP used form of address shows unawareness of cultural norms of respect, leaving the CALD resident feeling devaluated - HP underestimate residents’ cognitive abilities and the importance of shown respect |
